# Short Hairpin RNAs for Strand-Specific Small Interfering RNA Production

**DOI:** 10.3389/fbioe.2020.00940

**Published:** 2020-08-07

**Authors:** Peike Sheng, Krystal A. Flood, Mingyi Xie

**Affiliations:** ^1^Department of Biochemistry and Molecular Biology, University of Florida, Gainesville, FL, United States; ^2^UF Health Cancer Center, University of Florida, Gainesville, FL, United States; ^3^UF Genetics Institute, University of Florida, Gainesville, FL, United States

**Keywords:** RNA interference, short hairpin RNA, Argonaute, Dicer, microRNA, m^7^G-capped pre-miRNA, transcription start site miRNA

## Abstract

RNA interference (RNAi) is an effective mechanism for inhibiting gene expression at the post-transcriptional level. Expression of a messenger RNA (mRNA) can be inhibited by a ∼22-nucleotide (nt) small interfering (si)RNA with the corresponding reverse complementary sequence. Typically, a duplex of siRNA, composed of the desired siRNA and a passenger strand, is processed from a short hairpin RNA (shRNA) precursor by Dicer. Subsequently, one strand of the siRNA duplex is associated with Argonaute (Ago) protein for RNAi. Although RNAi is widely used, the off-target effect induced by the passenger strand remains a potential problem. Here, based on current understanding of endogenous precursor microRNA (pre-miRNA) hairpins, called Ago-shRNA and m^7^G-capped pre-miRNA, we discuss the principles of shRNA designs that produce a single siRNA from one strand of the hairpin.

## Introduction

Gene expression can be regulated at the post-transcriptional level in a number of ways. Powerful endogenous gene regulation mechanisms can be leveraged to effectively repair skewed pathways in diseased cells or tissues (Corbett, 2018). One example is the RNAi mechanism, in which small RNA-containing RNA-induced silencing complex (RISC) targets mRNA for translation inhibition or RNA degradation (Iwakawa and Tomari, 2015). Known as siRNAs, these small RNA molecules have sequences complementary to their target mRNAs. To produce siRNAs, shRNAs have been designed for targeted therapy in various conditions such as human immunodeficiency virus (HIV) type 1 infection; retinal disorders; and numerous cancers (Ahmadzada et al., 2018; Goguen et al., 2019; Massengill et al., 2019). However, deliveries into the cell and off-target effects remain among the largest issues afflicting the application of therapeutic shRNA (Dowdy, 2017). Despite these issues, shRNAs still appear to be more effective than small molecule drugs in various conditions, encouraging research to continue to develop shRNA systems modeled on endogenous miRNA biogenesis pathways (Lam et al., 2015).

miRNAs are endogenous 20–24 nt non-coding RNAs that are involved in post-transcriptional gene regulation. Most miRNAs are synthesized via the canonical biogenesis pathway. In this pathway, primary miRNA (pri-miRNA) is normally transcribed by RNA Polymerase II (Pol II), and contains a 7-methylguanosine (m^7^G) cap and a poly(A) tail. Drosha and DGCR8 (DiGeorge Syndrome Critical Region 8) form a nuclear Microprocessor complex to process the pri-miRNA into ∼70 nt precursor miRNA (pre-miRNA) hairpin (Denli et al., 2004; Han et al., 2006). Pre-miRNA is then exported by Exportin-5 (XPO5) into the cytoplasm to be cleaved by Dicer into ∼22 base pair (bp) mature miRNA duplexes (Yi et al., 2003; Lund et al., 2004; Macrae et al., 2006). Finally, miRNA duplexes are loaded onto Ago to activate RISC. Out of the two strands of the duplex, the strand that is preferentially incorporated in RISC is originally termed the miRNA strand or the guide strand. The complementary strand, which is ejected from RISC, is called the miRNA^∗^ strand or the passenger strand (Wang et al., 2009). However, subsequent studies have revealed potent miRNA^∗^/passenger strand activity in different conditions (Chen et al., 2018; Zhang et al., 2019). Therefore, current nomenclature designates the two strands as the 5p- or 3p- miRNA, depending on the origin of the pre-miRNA hairpin arms.

In mammals, a notable alternative miRNA biogenesis pathway produces transcription start site (TSS-) miRNAs. The precursor hairpin of a TSS-miRNA is transcribed with an m^7^G-cap on the 5’ end, making the biogenesis of TSS-miRNA independent of Drosha and XPO5 (Xie et al., 2013; Sheng et al., 2018). Another alternative miRNA biogenesis pathway skips the Dicer processing of pre-miRNA (Cheloufi et al., 2010; Cifuentes et al., 2010). Instead, this so-called “Ago-shRNA” is sliced by Ago2 and further trimmed into mature miRNA. So far only miR-451 is found to be produced via this pathway. Both alternative miRNA biogenesis pathways give rise to a functional miRNA from only one strand of the hairpin. In this article, we will discuss the principles of shRNA design, with a special emphasis on the two shRNAs that are capable of strand-specific siRNA production.

## Common shRNA Expression Methodology

Compared with pre-miRNAs, shRNAs are exogenous RNA molecules that can utilize the endogenous miRNA processing machineries to make small RNAs. The expression of shRNAs is commonly driven by either RNA Pol II or Pol III promoters and follow the canonical miRNA biogenesis pathway to be processed into siRNAs. For completeness, these strategies will be described briefly, as they were summarized in detail in a recent review (Bofill-De Ros and Gu, 2016).

**FIGURE 1:**
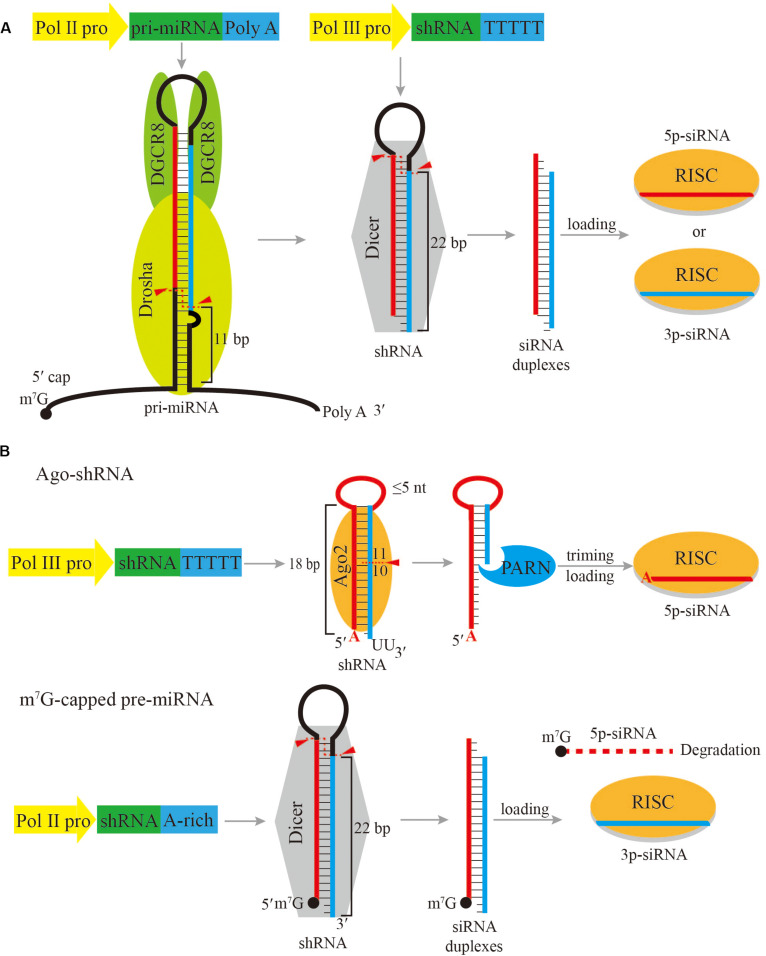
Schematic of shRNA expression cassettes and processing pathways. **(A)** Common shRNA expression cassettes driven by a Pol II promoter or a Pol III promoter, and the subsequent processing pathway. **(B)** Ago-shRNA and m^7^G-capped pre-miRNA expression cassettes and processing pathways. Ago-shRNA is cleaved by Ago2 and subsequently trimmed by PARN. The 5p-siRNA is loaded into RISC. m^7^G-capped pre-miRNA is cleaved by Dicer and the 3p-siRNA is loaded into RISC. Triangles indicate cleavage sites.

The most widely used strategy is to express shRNAs directly as Dicer substrate, while the gene targeting siRNA is located in either the 5p-arm or the 3p-arm of the shRNA. In this case, shRNAs are typically transcribed by RNA Pol III under the control of a U6 or H1 promoter (Brummelkamp et al., 2002; Sui et al., 2002; Figure 1A). The transcription initiation by the U6 promoter is more accurate and robust with a preference for purine as the TSS nucleotide. On the contrary, the H1 promoter can initiate transcription with either a purine or a pyrimidine, but the selection of its TSS is imprecise when it initiates with pyrimidine (Ma et al., 2014a; Herrera-Carrillo et al., 2017a). In order to accurately express shRNA, the first nucleotide of shRNA should be perfectly aligned with the TSS. Therefore the first nucleotide of shRNA driven by Pol III promoter should be either a guanosine (G) or an adenosine (A). In addition, Pol III recognizes a track of five or more thymidines (T) in the DNA template as the transcription termination signal, and automatically adds two uridines (U) at the 3’ end of the final transcript (Braglia et al., 2005). As a result, Pol III initiates transcription at a precise position starting with a purine and ends with two additional Us to produce shRNA mimicking the structure of a pre-miRNA, which has a 2-nt overhang at the 3’ end. Following transcription, the shRNA hairpin exits the nucleus with the assistance of XPO5, and can be further processed by Dicer into ∼22 bp siRNA duplexes following the 5’-end, 3’-end and the loop- counting rules similar to a pre-miRNA (Park et al., 2011; Gu et al., 2012; Tian et al., 2014).

Due to strong transcriptional activity of Pol III, Pol III promoter-driven shRNAs can exhibit toxicity arisen from competition with endogenous miRNAs (Grimm et al., 2006). Another way of expressing shRNA is to use weaker Pol II promoters, but the interference effect could also be reduced. Unlike Pol III promoter-driven constructs, most Pol II promoter-driven constructs express a long primary transcript, which needs to be processed by Drosha to produce the shRNA (Figure 1A). The primary transcript harboring the shRNA is essentially the same as an endogenous pri-miRNA. The primary transcript is processed into shRNA by the Microprocessor, which is composed of one Drosha molecule and two DGCR8 molecules. DGCR8 interacts with the apical loop and stem by its heme-binding domain and dsRNA-binding domains, respectively, allowing efficient and accurate processing (Nguyen et al., 2015; Kwon et al., 2016; Jin et al., 2020; Partin et al., 2020). As a result, the Microprocessor measures 10−11 bp from the basal single-stranded RNA and double-stranded (ds)RNA junction of the primary transcript and cleaves to release the shRNA. The advantage of Pol II promoters is that the expression of shRNA can be customized with more versatile regulatory elements, and multiple shRNAs can be expressed as a cluster on a single transcript to produce multiple siRNAs (Choi et al., 2015). Although Pol II promoter-driven constructs have several benefits, it is challenging to design an optimal primary transcript due to the incomplete understanding of the complex processing mechanism by the Microprocessor. At present, most Pol II promoter-driven constructs are designed based on endogenous pri-miRNA backbones, such as pri-miR-30a and pri-miR-155 (Stegmeier et al., 2005; Shan et al., 2009; Fellmann et al., 2013). As mechanistic studies of Microprocessor reveal further pri-miRNA processing details, more knowledge will be available to design optimized Pol II promoter-driven primary transcripts for siRNA expression.

## shRNA Designs for Strand-Specific siRNA Production

After Dicer precisely cleaves shRNAs into mature siRNA duplexes of about 22 nucleotides in length, thermodynamic properties determine which strand of the siRNA duplexes will be selected as the guide strand (Khvorova et al., 2003; Schwarz et al., 2003). shRNAs are designed based on this rule so that the desired siRNA is preferred by Ago. However, although to a lesser extent, the passenger strand can also be loaded into Ago and cause potential off-target effects (Bofill-De Ros and Gu, 2016). Recent researches have uncovered two miRNA biogenesis pathways that generate a single miRNA without a passenger strand. These pre-miRNAs are termed Ago-shRNA and m^7^G-capped pre-miRNA.

## siRNA Derived From Ago-shRNA

In vertebrates, the only miRNA that is derived from an Ago-shRNA is miR-451. In the miR-451 biogenesis pathway, after Drosha cleavage, the pre-miR-451 hairpin stem is only 17 bp long and contains a 4 nt loop. Therefore, pre-miR-451 is too short to be recognized by Dicer and is directly bound by Ago2. Ago2 precisely cleaves pre-miR-451 between the 10th and 11th base pairs in the 3p arm of the hairpin to generate an ∼30 nt extended intermediate small RNA (Cheloufi et al., 2010; Cifuentes et al., 2010; Herrera-Carrillo and Berkhout, 2017a). Subsequently, polyA-specific ribonuclease (PARN) trims the 3’ end of this small RNA to create the ∼22–26 nt mature miR-451 (Figure 1B; Yoda et al., 2013). Based on miR-451 biogenesis, an engineered Ago-shRNA can yield a siRNA from the 5p arm, which can avoid off-target effects that would be introduced by the passenger strand of a regular shRNA.

Currently, Ago-shRNA expression constructs normally contain a U6 promoter, similar to Dicer-dependent shRNAs described above. Therefore, the 5’ terminus of the Ago-shRNA is important (Herrera-Carrillo et al., 2017a). As discussed above, for a Pol III expression cassette, a purine as the 5’-terminal nucleotide ensures the accuracy of start-site selection and boosts transcriptional efficiency. In contrast, when a cytidine (C) or U, is on the 5’-terminal nucleotide of Ago-shRNA, the resulting siRNA activity is significantly reduced or even abrogated, compared to Ago-shRNAs with A or G as the 5’-terminal nucleotide. In addition, as the 5’ nucleotide of the siRNA, A is favored over G, presumably due to better interaction with the MID domain of Ago2 (Frank et al., 2010). In some cases, a mismatch at the bottom of the Ago-shRNA stem leads to higher siRNA activity. The precise mechanism of this preference is not clear. One possibility is that a strong base pair at the bottom of the hairpin will lead to partial shift from Ago2 to Dicer processing, because shRNA with greater stability is favored by Dicer (Herrera-Carrillo et al., 2017a).

Two important factors that set Ago-shRNA apart from Dicer-dependent shRNAs are the stem length and the loop size. If the stem of an shRNA is longer than 20 bp, the shRNA is processed by Dicer. If the stem of an shRNA is 19−20 bp, Dicer and Ago2 compete to process the shRNA. For an shRNA containing a stem between 16 and 18 bp, it is only processed by Ago2. shRNAs shorter than 16 bp will not be processed by Dicer or Ago2 into siRNAs, and thus lose all RNAi activity (Ge et al., 2010; Herrera-Carrillo et al., 2014; Ma et al., 2014b). On the other hand, shRNAs with a short stem but a large loop (>7 nt) will lead to a partial shift from Ago2 to Dicer processing, possibly due to steric hindrance between the loop with the PAZ domain of Ago2 (Wang et al., 2008). As a result, small loops of ≤5 nt are required for optimal Ago2-mediated processing (Liu et al., 2013).

In conclusion, the rule of thumb for designing a potent Ago-shRNA is to have an A as the 5’-terminal nucleotide, a stem of 18 bp, and a small loop (<5 nt). The optimal shRNA for expressing a specific siRNA should be experimentally tested based on the principles described above. In addition, similar to a Dicer-dependent shRNA, Ago-shRNA could be engineered into a primary transcript that requires Drosha processing (Kaadt et al., 2019), but the principle of processing a primary transcript of Ago-shRNA is complicated. For example, recent research shows that although pri-miR-451 is dependent on Microprocessor, its short stem and small terminal loop render it an intrinsically weak Microprocessor substrate. As a result, miR-451 requires miR-144, its close neighbor located only ∼100 bp away, as a helper to recruit and transfer Microprocessor locally to aid its biogenesis, although the identity and orientation of this neighbor are flexible (Fang and Bartel, 2020; Shang et al., 2020). Thus, it is possible that a primary transcript of Ago-shRNA needs to reside within a miRNA cluster for normal biogenesis, and more studies are needed to summarize general guidelines for this strategy. Nonetheless, Ago-shRNAs have been successfully implemented to knock down the human immunodeficiency virus 1 (HIV-1) RNA and the CCR5 co-receptor for HIV-1 infection (Herrera-Carrillo and Berkhout, 2017b; Herrera-Carrillo et al., 2017b), suggesting a promising future for Ago-shRNA therapeutics.

## siRNA Derived From m^7^G-Capped Pre-miRNA

In the biogenesis pathway for TSS-miRNAs, the pre-miRNAs are directly transcribed by Pol II with a m^7^G-cap placed at its 5’ ends (Xie et al., 2013; Zamudio et al., 2014; Sheng et al., 2018). These m^7^G-capped pre-miRNAs are therefore produced independently of Drosha processing. Subsequently, the capped pre-miRNA is exported from the nucleus to the cytoplasm preferentially by Exportin-1 (XPO1) and processed by Dicer to form a mature miRNA duplex. Afterwards, only the 3p-miRNA can be efficiently loaded onto Ago, while the 5p-miRNA is excluded from Ago because of the bulky m^7^G-cap (Figure 1B).

**FIGURE 2:**
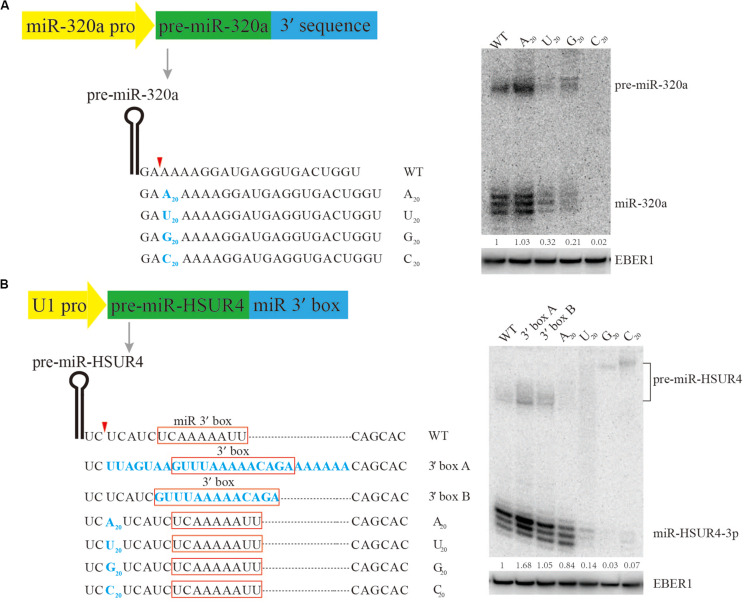
Mutational analysis of the 3’ end formation signal for m^7^G-capped pre-miRNA. Serial mutations were made downstream of pre-miR-320a **(A)** and pre-miR-HSUR4 **(B)**. Northern blotting was used to analyze the siRNA produced from each construct when transfected in HEK293T cells. Red boxes indicate the 3’ box sequence of HSUR4 or the miR 3’ box sequence of pre-miR-HSUR4. EBER1 is an Epstein-Barr virus encoded Pol III transcript serving as a transfection and loading control. Quantitation of relative mature miRNA levels is derived from two independent experiments.

It is known that the 5’ terminus of the m^7^G-capped pre-miRNA is produced directly by Pol II transcription initiation, but little is known about how it is terminated, making it difficult for practical design of m^7^G-capped pre-miRNAs for siRNA expression. Previous research has shown that pre-miR-320a is a m^7^G-capped pre-miRNA (Xie et al., 2013). To study the sequence requirement for m^7^G-capped pre-miRNA termination, plasmids are engineered to express pre-miR-320a driven by its own Pol II promoter, followed by various sequences to its 3’ end (Figure 2A). Since the native sequence downstream of pre-miR-320a is a conserved A-rich tract (Xie et al., 2013), we examined how consecutive nucleotides of A, U, G, or C would affect miR-320a generation. To this end, plasmids were generated to express pre-miR-320a followed immediately by a 20-nt insertion comprised of only A, U, G, or C. After transfection of the plasmids into HEK 293T cells, Northern blotting was performed to examine miR-320a levels. The results show that an A_20_ insertion can produce slightly more miR-320a than other insertions, at an expression level comparable to that of the wild-type (WT) (Figure 2A). Therefore, an A-rich sequence is recommended as the 3’ end termination sequence for expressing a m^7^G-capped pre-miRNA.

To further confirm that an A-rich sequence acts as a universal terminal sequence for Pol II, a strong U1 promoter, which can also be used to express m^7^G-capped pre-miRNA, was subjected to similar tests. Previously, we have used the U1 promoter to express miR-HSUR4, a viral miRNA from *Herpesvirus saimiri* (HVS) (Xie et al., 2015). In HVS, pre-miR-HSUR4 is processed from a primary transcript by the Integrator complex, bypassing Drosha cleavage. Downstream of pre-miR-HSUR4, an A-rich sequence called “miR 3’ box” recruits the Integrator complex for 3’ end processing. Here, we construct plasmids with pre-miR-HSUR4 immediately downstream of the U1 promoter, and made various insertions upstream of the miR 3’ box to test how they can influence miR-HSUR4 expression (Figure 2B). Similarly, 20 consecutive nucleotides of A, U, G, or C, were inserted. Albeit slightly less efficiently than the WT miR 3’ box, the plasmids containing an A_20_ insertion produced the largest amount of miR-HSUR4 compared to other sequences. This set of constructs also demonstrates that the distance between pre-miRNA and the miR 3’ box is important. With C_20_/U_20_/G_20_ insertions before the miR 3’ box, Integrator cleavage upstream of the 3’ box produces 3’ extended pre-miRNAs (Figure 2B). However, these 3’ extended pre-miRNAs cannot be processed by Dicer, leading to abrogated miR-HSUR4 production. We also tested the effect of replacing miR 3’ box with the 3’ box, which is the signal for 3’ end processing of snRNAs by Integrator. The 3’ box of HSUR4 (HVS U rich RNA 4) snRNA and the surrounding sequences were used (Cazalla et al., 2011). The results show that replacing the miR 3’ box with the 3’ box including the surrounding sequences leads to the highest levels of miR-HSUR4 (Figure 2B). Taken together, for the expression of a potent shRNA with a m^7^G-cap, it is optimal to terminate with an A-rich sequence, especially a sequence resembling the snRNA 3’ box when the U1 promoter is used.

## Future Prospects and Conclusion

Although RNAi has already been used in clinical practice, its off-target effect caused by the passenger strand greatly hinders broader application. To overcome this obstacle, two shRNA designs can be used to produce a single 5p- or 3p-siRNA, respectively, according to studies of endogenous miRNA maturation pathways. These alternative miRNA biogenesis pathways bypass one or more of the canonical miRNA biogenesis factors. The Ago-shRNA pathway is Dicer-independent and critically involved in the biogenesis of miR-451. The m^7^G-capped pre-miRNA pathway bypasses Drosha processing and can employ XPO1 to subsequently export the pre-miRNA from the nucleus to the cytoplasm. Therefore, producing siRNAs from these two alternative pathways would also reduce the indirect off-target effects that result from competing with the bulk of endogenous miRNAs for limited biogenesis factors.

Previous studies have shown that precise processing of the shRNA is critical to gene silencing efficiency and target specificity (Gu et al., 2012; Denise et al., 2014; Ma et al., 2014a). A traditional shRNA expression cassette uses a U6 promoter and terminates the 3’ end by a poly(U) tail. The 5’- and 3’- ends of Ago-shRNA are also achieved in this way when expressed from the U6 promoter (Bofill-De Ros and Gu, 2016). To produce a m^7^G-capped shRNA, a Pol II promoter such as the U1 promoter needs to be used. Although the initiation nucleotides and the surrounding sequences for expressing a shRNA have not been systematically studied, a purine is presumably required. In addition, m^7^G-capped shRNA with 5’-extension can be processed by Dicer (Sheng et al., 2018), inviting future research to analyze the length and the composition of 5’-extension for optimal siRNA expression. The termination mechanism for a m^7^G-capped shRNA expressed from a non-snRNA Pol II promoter is not clear (Xie et al., 2013). For example, the endogenous miRNA-320a has heterogeneous 3’ end which is encoded by the genomic sequence, indicating that their 3’-end termination may be imprecise. A more precise 3’ end formation of the m^7^G-capped shRNA may be achieved when expressed from the U1-promoter and relies on the Integrator complex for 3’ end cleavage. The 3’ box of HSUR4 and the miR 3’ box of pre-miR-HSUR4 can be specifically recognized by the Integrator complex. However, heterogenous siRNA length is still evident on the Northern blot (Figure 2), which may result from either imprecise 3’ end cleavage, or from 3’ end modification on the mature siRNA. Recently, a self-cleaving ribozyme derived from the hepatitis delta virus has been introduced to enhance the precision of 3’ end cleavage of shRNAs, which may solve the problem of imprecise termination of shRNA 3’ ends (Shang et al., 2015).

Although RNAi plays an important role in post-transcriptional level regulation of gene expression, it has been greatly challenged by the development of CRISPR-Cas technology (Cong et al., 2013; Barrangou and Doudna, 2016; Wang et al., 2020). CRISPR-Cas9 has tremendous potential in gene editing therapy. However, as a mature technology, RNAi still has its unique advantages. Catalytically active CRISPR-Cas9 edits a gene to inhibit gene expression, in which the resulting DNA mutation or chromosome rearrangements are permanent. Since the deletion of essential genes can be fatal, their functions should be studied by RNAi, which only partially reduces the expression level of the gene. Recently, the newly discovered CRISPR-Cas13 can regulate gene expression at the RNA level in a way similar to RNAi, expanding the utility of the CRISPR-Cas system (Abudayyeh et al., 2017; Cox et al., 2017). Nonetheless, RNAi is achieved using endogenous protein machinery, while CRISPR requires the introduction of the exogenous Cas proteins, which have the potential to cause deleterious immunogenicity (Crudele and Chamberlain, 2018). Therefore, the two technologies have their own advantages and complement each other in regulating gene expression.

## Data Availability Statement

The datasets presented in this article are not readily available. Requests to access the datasets should be directed to mingyi.xie@ufl.edu.

## Author Contributions

PS and MX conceived the project and were involved in experiments. PS, KF, and MX wrote the manuscript. All authors contributed to the article and approved the submitted version.

## Conflict of Interest

The authors declare that the research was conducted in the absence of any commercial or financial relationships that could be construed as a potential conflict of interest.
